# Pharmacophore Generation from a Drug-like Core Molecule Surrounded by a Library Peptide via the 10BASE_d_-T on Bacteriophage T7

**DOI:** 10.3390/molecules19022481

**Published:** 2014-02-21

**Authors:** Yuuki Tokunaga, Yuuki Azetsu, Keisuke Fukunaga, Takaaki Hatanaka, Yuji Ito, Masumi Taki

**Affiliations:** 1 Department of Engineering Science, Bioscience and Technology Program, The Graduate School of Informatics and Engineering, The University of Electro-Communications (UEC), 1-5-1 Chofugaoka, Chofu, Tokyo 182-8585, Japan; E-Mails: t1333065@edu.cc.uec.ac.jp (Y.T.); a1013005@edu.cc.uec.ac.jp (Y.A.); fukunaga-ks@pc.uec.ac.jp (K.F.); 2 Department of Chemistry and Bioscience, Graduate School of Science and Engineering, Kagoshima University, 1-21-35 Korimoto, Kagoshima, Kagoshima 890-0065, Japan; E-Mails: k7808291@kadai.jp (T.H.); k2174603@kadai.jp (Y.I.)

**Keywords:** 10BASE_d_-T, bacteriophage T7, chemical modification, drug-like molecule, peptide library, phage display, pharmacophore, salicylic acid, site-specific conjugation, thioether

## Abstract

We have achieved site-specific conjugation of several haloacetamide derivatives into designated cysteines on bacteriophage T7-displayed peptides, which are fused to T7 capsid protein gp10. This easiest gp10 based-thioetherification (10BASE_d_-T) undergoes almost quantitatively like a click reaction without side reaction or loss of phage infectivity. The post-translational modification yield, as well as the site-specificity, is quantitatively analyzed by a fluorescent densitometric analysis after gel electrophoresis. The detailed structure of the modified peptide on phage is identified with tandem mass spectrometry. Construction of such a peptide-fused phage library possessing non-natural core structures will be useful for future drug discovery. For this aim, we propose a novel concept of pharmacophore generation from a drug-like molecule (*i.e.*, salicylic acid) conjugated with surrounding randomized peptides. By using the hybrid library, streptavidin-specific binders are isolated through four rounds of biopanning.

## 1. Introduction

Recently, post-translational chemical modification of bacteriophage (referred to hereafter as phage) -displayed peptides is attracting attention for drug discovery [1,2,3]. A pioneering work in the field of non-natural peptide/protein library construction on phage was reported in 2004; a fluorogenic biosensor was developed by conjugating a fluorophore with a designated Cys at the antigen-binding site of antibody library on the phage [4]. Until now, multiple research groups have reported construction of non-natural peptide libraries by the post-translational chemical modifications of M13 phage-displayed peptides [5,6,7,8,9,10]. Another type of phage, T7, is also used for phage display [11,12], and is superior to the M13 phage in terms of bias and handling [13]. Very recently, we have constructed a non-natural peptide library by the post-translational chemical modification of T7 phage-displayed peptides, namely gp10 based-thioetherification (10BASE_d_-T) [14]. The 10BASE_d_-T is carried out in one-pot without side reactions or loss of phage infectivity; the reaction efficiency and site/position specificity of the 10BASE_d_-T on the T7 phage were as excellent as those of (chemo)enzymatic or click introduction of functional groups on proteins. By using a tetramethylrhodamine (TMR)-conjugated library via the 10BASE_d_-T, glutathione *S*-transferase specific-binders have been discovered. 

**Figure 1:**
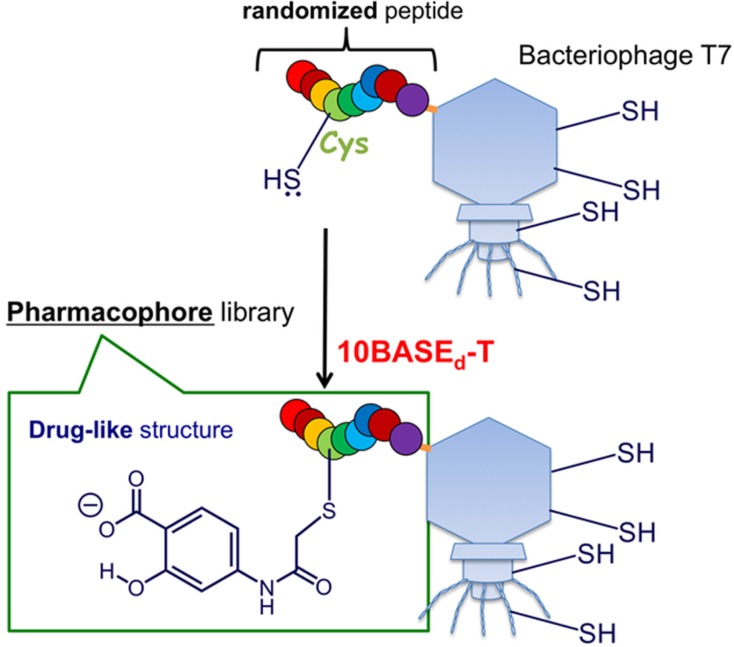
Construction of a peptide-fused pharmacophore library with a model drug-like (*i.e.*, salicylic acid; Sal) core structure via the 10BASE_d_-T.

A potential limitation of this library is that the TMR moiety is too bulky and hydrophobic, which may cause non-specific binding or aggregation of the binders. Thus, we envisioned that conjugation of a water-soluble and small drug-like molecule, instead of TMR, to a peptide library would increase the possibility to discover target-specific binders. Here we attempt a novel concept of a pharmacophore generation by conjugation of a drug-like core molecule to a surrounding randomized library peptide via the 10BASE_d_-T (Figure 1). As the model drug-like core molecule, commercially available 4-iodoacetamidosalicylic acid (Sal-IA) was used because it is one of the smallest pharmaceutical molecules possessing both hydrophobic benzene ring and hydrophilic hydroxyl/carboxyl groups. These groups may potentially interact with various proteins through hydrophobic/π-interactions and hydrogen bonding [15], and seldom form aggregates [16]. If surroundings of the drug-like core molecule are optimized for interaction with target biomolecules of interest, novel specificity and affinity would be generated. Thus, we constructed an artificial library of salicylic acid (Sal) possessing randomized peptides via the 10BASE_d_-T.

## 2. Results and Discussion

### 2.1. Optimization and Identification of Site-Specific Introduction of Acetamidosalicylic Acid via the 10BASE_d_-T

**Figure 2:**
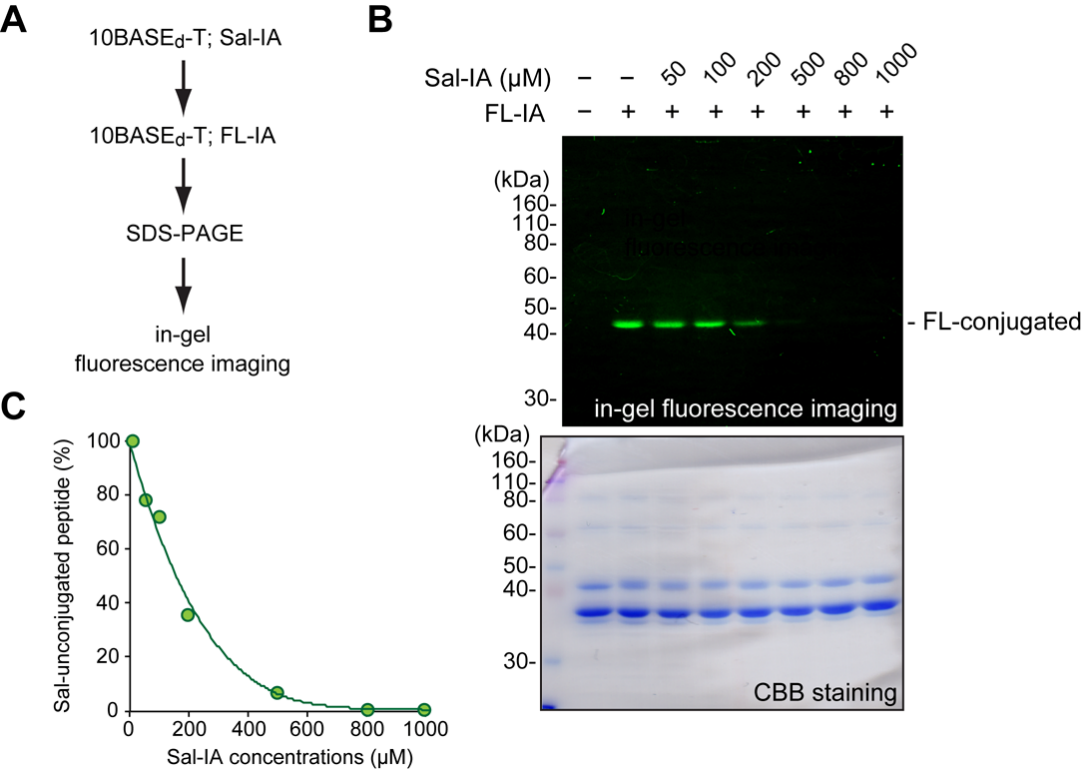
Optimization of Sal-IA concentration. (**A**) Procedure of the optimization. A T7 phage-displayed model peptide was modified via the 10BASE_d_-T with various molar concentrations of Sal-IA under standard conditions (described in the Experimental). After the Sal-conjugation, the peptide was further modified via the 10BASE_d_-T with 200 μM of FL-IA. Equal amounts of phage proteins were subjected to SDS-PAGE followed by fluorescence imaging. Sal-IA and FL-IA represents salicylic acid- iodoacetamide and fluorescein-iodoacetamide, respectively. (**B**) In-gel fluorescence imaging after the gel electrophoresis (upper panel). Total T7 phage proteins were stained with Coomassie brilliant blue (CBB) (lower panel). (**C**) Percentages of Sal-unconjugated peptide in each concentration of Sal-IA.

For the optimization of site-specific introduction of Sal group via the 10BASE_d_-T, we mixed Sal-IA with various molar concentrations and a T7-displayed model peptide (1.0 × 10^11^ plaque forming units of the T7 phage) in 700 μL of phosphate-buffered saline (pH 7.4) supplemented with 500 μM tris(2-carboxyethyl)phosphine (TCEP) and 400 mM NaCl at 4 °C. After 3 h reaction, the peptide was further treated with 5-idodoacetamide-fluorescein (FL-IA; 200 μM) for 3 h at 4 °C. The latter reaction with fluorescent FL-IA blocks all the unreacted SH-groups on the T7 phage-displayed peptide after the conjugation with Sal-IA. Our previous study demonstrated that fluorescent tetramethylrhodamine-iodoacetamide is conjugated to at least 95% of T7 phage-displayed peptide [14]. Similarly, we confirmed that FL-IA was conjugated to at least 95% of the displayed peptide when 200 μM of FL-IA was used for the modification (data not shown). Thus, we can indirectly estimate conversion yield of the Sal-IA conjugation by fluorescent densitometric analysis (Figure 2A). After the 10BASE_d_-T, whole T7 phage proteins were subjected to sodium dodecyl sulfate poly-acrylamide gel electrophoresis (SDS-PAGE), followed by in-gel fluorescence imaging. When Sal-IA was absent in the reaction mixture, a single fluorescent band could be seen at an appropriate molecular weight (*ca.* 44 kDa) of the peptide-fused gp10 (Figure 2B; upper panel, lane 2). This indicates that the alkylation with FL-IA exclusively occurred at the peptide-fused gp10. Note that neither protein components for the infection nor other T7 phage molecules were included in the reaction [14]. When the concentration of Sal-IA was increased, the fluorescent band disappeared. This indicates that the designated Cys on the displayed peptide had already reacted with Sal-IA, and FL-IA no longer reacted with the peptide. From the densitometric analysis, the optimal molar concentration of Sal-IA was around 800 μM; almost all the designated Cys on the peptide reacted with Sal-IA (Figure 2C). Thus, we successfully established a quantification method of the 10BASE_d_-T reaction in a visible manner, even if the introduced non-natural molecule does not possess any chromophores. Recently, Derda and co-workers demonstrated a compatible quantification method, namely a biotin capture assay, to determine the chemical modification yield on M13 phage-displayed peptides [6,10]. In this method, biotin is once conjugated to the M13 phage-displayed peptides. Then, the biotin-conjugated M13 phage is captured by streptavidin-conjugated affinity beads and subjected to a plaque assay. This method might be useful when the amount of the modified phage is extremely limited. If it is sufficient, our quantification method shown above is rapid and straightforward.

**Figure 3:**
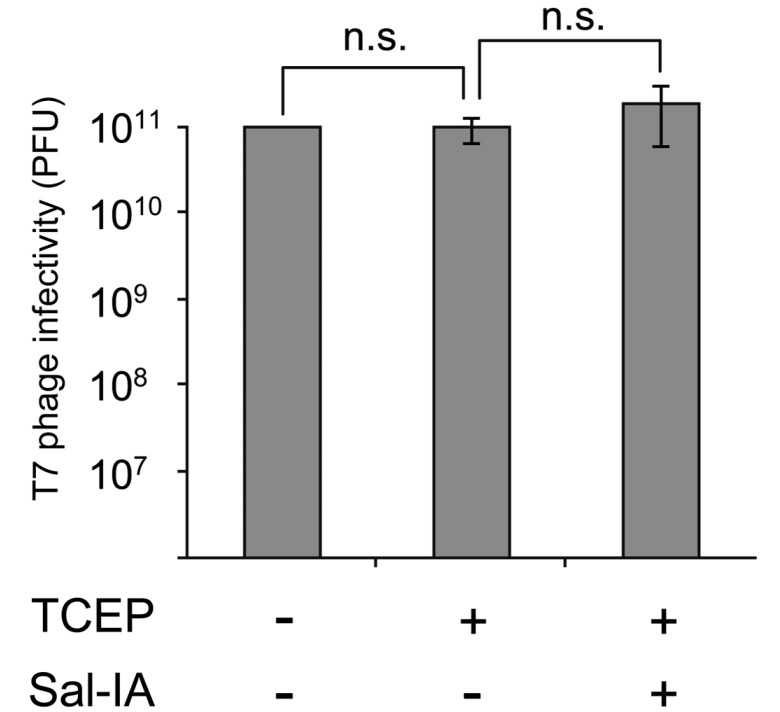
Infectivity of Sal-conjugated T7 phage library. A T7 phage library (-S-G-G-G-X_3_-C-X_6_-C-X_3_; X represents any randomized amino acid) was treated with or without salicylic acid-iodoacetamide (Sal-IA; 800 μM) in the presence of TCEP (500 μM) under standard conditions (see Experimental Section). The number of plaque forming units (PFU) was determined by a serial dilution method and plaque assay. The graph summarizes the results of three independent experiments. Error bars represent standard deviations. Statistical analysis was performed by unpaired Student’s *t*-test. n.s., not significant (*p* values = 0.40 [left] and 0.22 [right], respectively).

Under the optimized modification conditions, we examined the infectivity of the Sal-conjugated T7 phage peptide library by plaque assay and found that the modified T7 phage retained its infectivity (Figure 3). This suggests that the T7 phage is an excellent platform for post-translational modifications.

**Figure 4:**
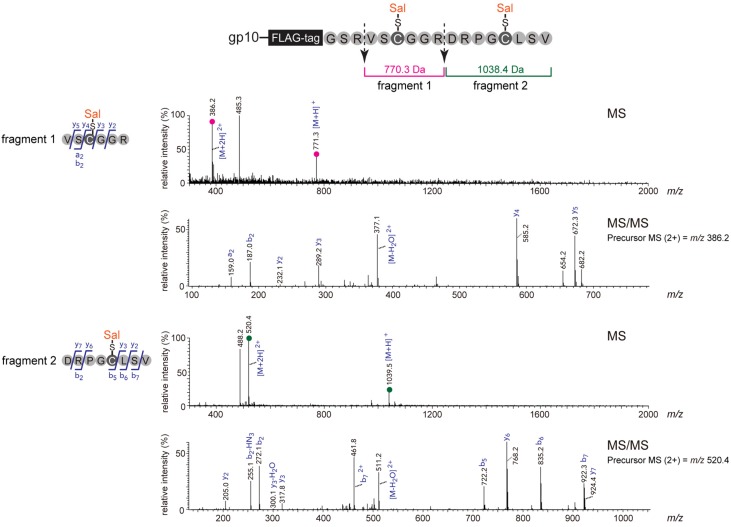
Introduction of Sal onto T7 phage-displayed peptide via the 10BASE_d_-T. Here, a model T7 phage carrying a high-copy vector was used. After the 10BASE_d_-T followed by SDS-PAGE, Sal-conjugated peptide-fused gp10 was excised and subjected to in-gel trypsinization followed by conventional LC-MS/MS analysis. Note that trypsin could not cleave before proline [14]. MS and MS/MS spectra correspond to the trypsinized two peptide fragments possessing the Sal moiety.

To achieve more precise characterization, we performed a mass spectrometric analysis of the Sal-conjugated peptide. MS analysis of the phage-displayed peptide is challenging because small amounts of peptides are obtained per batch [2]. Similar to M13 phage-displayed peptides fused to a minor coat protein pIII [17], relatively few copies (*ca.*, 10 molecules) of peptides are displayed per single T7 phage virion when a mid-copy phage display vector (T7Select10) is used [18]. These protein amounts are nearly at the detection limit for MS-based characterization of the peptide [2]. To bypass this problem, we generated a model T7 phage [14] carrying approximately 200 molecules of the peptides per single virion by using a high-copy vector (T7Select415) [19]. In our previous study, we successfully analyzed TMR-conjugated peptides on the model T7 phage by conventional LC-MS/MS system [14]. After the 10BASE_d_-T with Sal-IA followed by SDS-PAGE, the peptide-fused gp10 band around 40 kDa in the gel was excised and digested with trypsin. The resulting peptide fragments were analyzed by LC-MS/MS. Two chromatographic peaks at 300-400 nm (Sal absorption) were detected (data not shown), and each of them was identified as the Sal-conjugated T7 phage-displayed peptide by tandem mass spectrometry (Figure 4). Also these data suggest that two Sal molecules were conjugated to the two designated Cys on T7 phage-displayed peptides. At the same time, most of the other peptide fragments derived from gp10 were identified by peptide mass fingerprinting based on MS/MS ion search (data not shown). Very recently, mass spectrometric analysis of the modified M13 phage-displayed peptide was reported by using high-performance MS systems [20,21]. In future studies, not only qualitative but also quantitative mass spectrometric study of the modified phage-displayed peptide might be available.

### 2.2. Construction of Peptide-Conjugated Acetamidosalicylic Acid Library and Selection of a Streptavidin-Specific Binder

**Figure 5:**
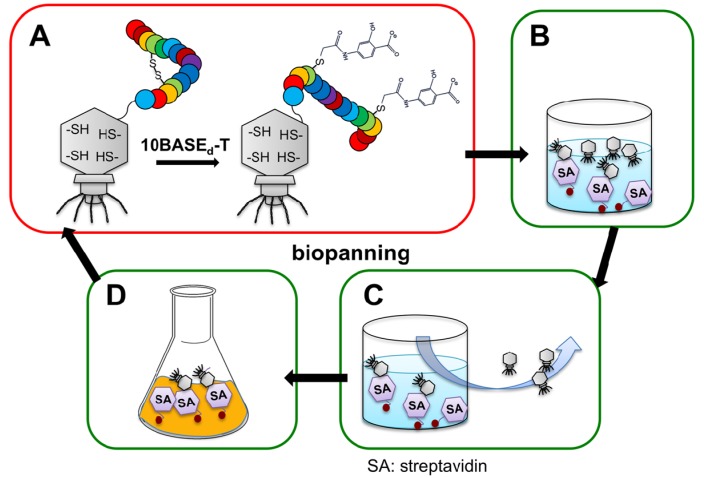
Schematic diagram of biopanning used in this study. (**A**) Construction of peptide-conjugated Sal library on T7 phage through the 10BASE_d_-T. (**B**) Incubation of the phage display library with immobilized streptavidin. (**C**) Washing of unbound phage. (**D**) Amplification of the streptavidin-bound phage for subsequent rounds of biopanning.

**Figure 6:**
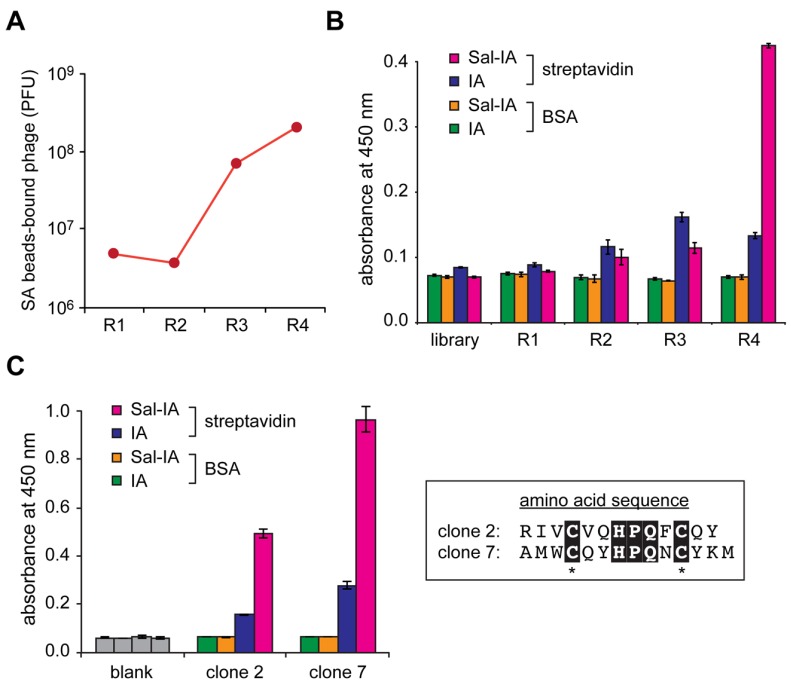
(**A**) Enrichment of streptavidin (SA)-coupled beads-bound T7 phage through biopanning. PFU (plaque forming units) and R indicate the number of the beads-bound T7 phage and rounds of biopanning, respectively. (**B**) ELISA of modified T7 phage polyclones against streptavidin or bovine serum albumin (BSA). BSA was used as a negative control to examine non-specific binding. Sal-IA and IA indicate salicylic acid-iodoacetamide and iodoacetamide, respectively. Error bars represent standard deviations of three independent experiments. (**C**) ELISA of two types of T7 phage monoclones randomly chosen from the phage pool after 4 rounds of biopanning. The peptide sequences displayed on the phage are shown in the right panel.A consensus sequence is highlighted. An asterisk indicates the modified cysteine. For the alignment, ESPript program (http://espript.ibcp.fr/) [28] was used. A negative control experiment (blank) was also performed in the absence of T7 phage.

We next attempted to find target-specific binders from the peptide-conjugated Sal library as shown in Figure 5. In advance of biopanning, a T7 phage library carrying X_3_-C-X_6_-C-X_3_ peptides (where X represents any amino acid), which had been generated from the mid-copy vector (T7Select10), were modified through the 10BASE_d_-T. To include the possibility that double core structures may improve affinity toward the target protein, we conjugated two Sal-groups into a single library peptide. Four rounds of biopanning were performed against streptavidin as a model target protein. Enrichment of the streptavidin-binder was confirmed by plaque assay after the washing step in biopanning; the population of streptavidin-bound T7 phage was increased through biopanning (Figure 6A). Binding specificity to streptavidin was confirmed by enzyme-linked immunosorbent assay (ELISA). To examine whether the Sal but not the amide moiety is required for the binding, Sal-IA-treated and iodoacetamide (IA)-treated T7 phage were subjected to ELISA in parallel. After four rounds of biopanning, the Sal-conjugated T7 phage-displayed peptides showed the strongest binding to streptavidin (Figure 6B), suggesting that the Sal moiety of the peptides played a crucial role in interaction with the target. Eight of the T7 phage monoclones were randomly chosen from the phage pool. Three of the streptavidin-bound monoclones were subjected to DNA sequencing analysis, whereas five of the clones did not bind to streptavidin (data not shown) probably because four rounds of biopanning may not be sufficient to attain complete affinity maturation. Two of the positive clones had the same sequence (clone 7; -A-M-W-C-Q-Y-H-P-Q-N-C-Y-K-M), and one was different (clone 2; -R-I-V-C-V-Q-H-P-Q-F-C-Q-Y) (Figure 6C). In combination with our result from similar Sal-conjugated phage library carrying X_3_-C-X_9/10_-C-X_3_ peptides [22], we found that all of the positive clones had a consensus sequence of H-P-Q-X-C* (C* and X represent alkylated Cys and any amino acid, respectively) (Figure 6C, left panel), which contains the known streptavidin-binding sequence (HPQ) [23,24]. When compared with the IA-modified T7 phage-displayed peptide, the Sal-IA-modified peptide significantly bound to streptavidin. This means that the Sal moiety and the surrounding consensus peptide cooperatively enhanced the binding ability toward streptavidin, to generate a novel pharmacophore (Figure 6C).

**Figure 7:**
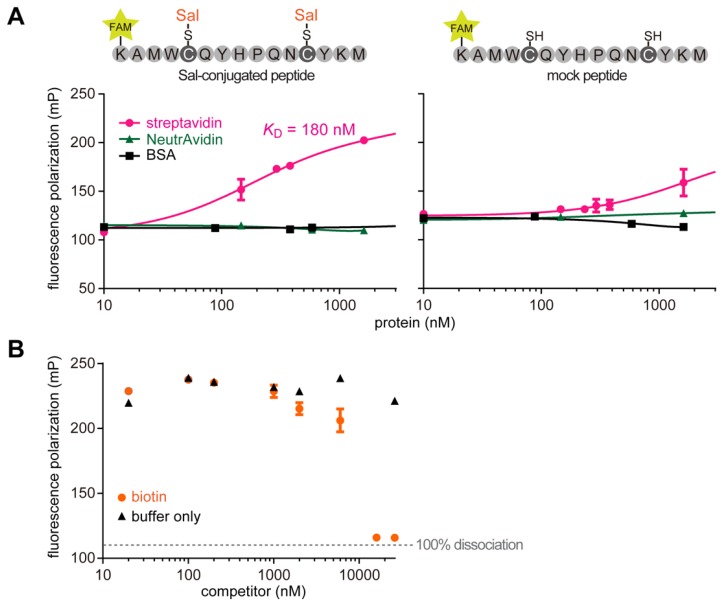
(**A**) Determination of streptavidin-binding affinities of the Sal-conjugated peptide (left panel) and the mock peptide (right panel) by fluorescence polarization assay. The amino acid sequence of the peptide is shown in the upper part. The plots indicate the polarization (mP) of the fluorophore (FAM)-coupled peptides in the presence of various concentrations of target proteins. NeutrAvidin and bovine serum albumin (BSA) were used as mock target proteins. (**B**) Competitive binding assay. Biotin as a competitor was mixed with the Sal-conjugated peptide-streptavidin complex. Error bars represent standard deviations.

To determine the binding affinity of the peptide to streptavidin by using a fluorescence polarization (FP) assay, we chemically synthesized a fluorescent peptide possessing the sequence of clone 7 (K-5/6-FAM-A-M-W-C*-Q-Y-H-P-Q-N-C*-Y-K-M-NH_2_; C* represents Sal-conjugated Cys). The synthesized linear peptide bound to streptavidin in a protein-specific manner (Figure 7A, left panel), which is consistent with a previous report that HPQ-containing peptide does not bind to non-glycosylated forms of chicken avidin (NeutrAvidin®) [25]. The dissociation constant (*K*_D_) was estimated to be 180 nM. Usually, linear peptides bind to a target with a lower affinity than cyclic ones, because the latter rigid structures minimize conformational entropy loss associated with the binding [13,26]. Indeed, HPQ-containing cyclic peptides bind to streptavidin with a higher affinity [23,27]. Nevertheless, the affinity of our Sal-conjugated linear peptide was almost the same as that of the HPQ-containing cyclic peptide [27]. On the other hand, mock (Sal-unconjugated) peptide bound to streptavidin with a roughly 10-fold lower affinity under reducing conditions (Figure 7A, right panel). This again suggests that the excellent pharmacophore was generated only when Sal was surrounded by the appropriate peptide.

HPQ-containing peptides are usually biotin-mimetics [23], thus we examined whether the Sal-conjugated peptide binds to streptavidin in the same manner. As expected, interaction of the Sal-conjugated peptide with streptavidin was fully disrupted by biotin (Figure 7B), suggesting that the peptide-conjugated Sal binds to the biotin-binding site of streptavidin.

## 3. Experimental

### 3.1. General

All experiments were performed with commercially available reagents and kits. Note that no special materials and skills are needed. Contrary to popular belief and what is stated in the T7Select system manual (Merck Millipore, Bedford, MA, USA), CsCl step gradient and ultracentrifugation are not necessary to perform all of the experiments to obtain a target-specific binder. For purification of T7 phage at every step by polyethyleneglycol/NaCl precipitation, we only used a conventional centrifugation system which can rotate at 13,000 rpm. In all phage display experiments, *Escherichia coli* BLT5403 cells were used. Note that medium-high-copied peptide display (200 peptides per virion) is produced by an amplification of T7 phage carrying a T7Select415-1b high-copy vector by using BLT5403 cells [14,19].

### 3.2. Construction of T7 Phage Display Libraries

A T7 phage display peptide library (-S-G-G-G-X_3_-C-X_6_-C-X_3_; X represents any amino acid) was constructed in the same manner of our reported method [11,12,14].

### 3.3. Chemical Modification of T7 Phage-Displayed Peptide via the 10BASE_d_-T

The procedures with standard reaction conditions of the 10BASE_d_-T are as follows: tris(2-carboxyethyl)phosphine (TCEP) and 4-iodoacetamidosalicylic acid (Sal-IA; cat. No. 102065, MP Biomedicals, Santa Ana, CA, USA) stock aqueous solutions of pH 7 should be prepared by neutralization with NaOHaq in advance. All reactions were carried out in 1.5 mL microcentrifuge tubes. T7 phage particles (approximately 1.0 × 10^11^ PFU) were resuspended in 700 μL of phosphate-buffered saline (PBS; cat. No. 14249-95, Nacalai tesque, Kyoto, Japan) supplemented with 400 mM NaCl, and well dissolved by vortexing. At this step, the solution contained 1.0 × 10^12^ (T7 phage made by T7Select10-3b system for the selection) or 2.0 × 10^13^ (by T7Select415-1b system for the optimization of Sal-IA concentration) molecules of the T7-displayed peptides. After centrifugation at 12,000 rpm for 5 min at room temperature, the supernatant was mixed with neutralized TCEP-NaOH (final concentration of 500 μM) at 4 °C. 

*For the selection against streptavidin:* Neutralized Sal-IA-NaOH was added to the above TCEP-treated phage at a final concentration of 800 μM, and the mixture was incubated at 4 °C for 3 h in the dark with shaking. To inactivate the unreacted Sal-IA, 2-mercaptoethanol was added to the mixture at a final concentration of 5 mM, and further incubated at 4 °C for several minutes. The T7 phage particles were precipitated with a mixture of polyethylene glycol 6000 (Nacalai tesque) and sodium chloride to final concentrations of 5% (w/v) and 0.5 M, respectively. After centrifugation, the precipitate was dissolved in an appreciate buffer.

*For the optimization of Sal-IA concentration:* Neutralized Sal-IA-NaOH was added to the above TCEP-treated phage at various concentrations, and the mixture was incubated at 4 °C for 3 h in the dark with shaking. After the reaction, 5-iodoacetamide-fluorescein (FL-IA) stock solution in dimethyl sulfoxide/water (1:1 by volume) was added at a final concentration of 200 μM, and the mixture was further incubated at 4 °C for 3 h. FL-IA was purchased from Sigma-Aldrich (cat. No. I9271, St. Louis, MO, USA). The latter reaction with fluorescent FL-IA reacts with all the *unreacted* SH-groups on the displaying peptide after the conjugation with Sal-IA. Thus, we can indirectly estimate conversion yield of the Sal-IA treatment by SDS-PAGE followed by densitometric fluorescence analysis of the peptide-fused gp10 as described in Section 3.4. Site-specificity of the 10BASE_d_-T by using FL-IA was confirmed by LC-MS/MS (supplementary Figure S1). For the optimization, a model T7 phage with medium-high-copied peptides possessing the -G-S-R-V-S-C-G-G-R-D-R-P-G-C-L-S-V sequence at the C-terminal region of gp10 was used [14].

### 3.4. In-Gel Fluorescence Imaging

T7 phage particles were dissolved in 1× sample buffer (62.5 mM tris(hydroxymethyl)-aminomethane-HCl, pH 6.8, 10% glycerol, 2% SDS, 5% 2-mercaptoethanol, 0.002% bromophenol blue). The solution was incubated at 95 °C for 5 min, and then subjected to SDS-PAGE. Proteins were resolved by a 10% polyacrylamide gel. After electrophoresis, the FL-conjugated proteins were visualized by in-gel fluorescence imaging using a conventional gel imager (ChemiDoc XRS+, Bio-Rad Laboratories, Hercules, CA, USA) excited with UV light. 

### 3.5. Mass Spectrometric Analysis

For mass spectrometric analysis, a PAGE gel was stained with Rapid Stain CBB kit (Nacalai tesque, cat No. 30035-14). The stained protein bands were excised from the gel. Proteins in the gel were reduced with 25 mM dithiothreitol at 65 °C for 10 min, and then alkylated with 55 mM iodoacetamide at room temperature for 60 min in the dark. Digestion was carried out with modified trypsin (Promega, Madison, WI, USA) at 37 °C overnight. The resulting peptides were analyzed using Agilent 1100 semi-micro HPLC system (Agilent Technologies, Santa Clara, CA, USA) equipped with a C_18_ reverse-phase column (Hypersil GOLD, 2.1 × 100 mm, Thermo Fisher Scientific, Waltham, MA, USA) connected to LCQ-Fleet mass spectrometer. The peptides were separated using a 0%–50% gradient of acetonitrile containing 0.1% formic acid during 40 min at a flow rate of 300 μL per minute, and then eluted peptides were directly sprayed into the mass spectrometer. The mass spectrometer was operated in the data-dependent mode and externally calibrated. Survey MS scans were acquired in the 300–2000 or 400–2000 *m/z* ranges. Multiply charged ions of high intensity per scan were fragmented with collision-induced dissociation in the ion trap. A dynamic exclusion window was applied within 30 s. All tandem mass spectra were collected using normalized collision energy of 40%. Data were acquired and analyzed with Xcalibur software v. 2.07 (Thermo Scientific). 

### 3.6. Biopanning against Streptavidin

For biopanning, approximately 8.4 × 10^10^ PFU of a T7Select10 library (-S-G-G-G-X_3_-C-X_6_-C-X_3_; X represents any randomized amino acid) were modified via the 10BASE_d_-T. The modified T7 library was dissolved in a selection buffer (PBS supplemented with 0.5% Triton X-100 and 1 mM TCEP-NaOH), and incubated with streptavidin-immobilized nanomagnetic beads [29] (FG streptavidin beads, cat No. TAS8848N1170, Tamagawa Seiki, Nagano, Japan) at 4 °C. The beads were washed three times with 200 μL of the selection buffer, and the streptavidin-bound phages were directly infected and amplified with *E. coli* BLT5403 cells. Stringent conditions were applied to each round by shortening the binding time and by increasing the washing frequency. For binding and washing, an automated bioscreening machine for phage display system (TargetAngler 8, Tamagawa Seiki, Nagano, Japan) was used. After four rounds of biopanning, randomly chosen T7 phage clones were subjected to DNA sequencing. 

### 3.7. Enzyme-Linked Immunosorbent Assay (ELISA)

830 pmol of streptavidin (Wako Pure Chemical Industries, Osaka, Japan) was dissolved in PBS, and immobilized on each well of a 96-well immunoplate (Nunc MaxiSorp, Thermo Scientific). After washing with PBS, surface of the wells were coated with 1% (w/v) BSA in PBS supplemented with 0.05% Tween-20 at 4 °C overnight. Approximately 2.0 × 10^10^ PFU of the T7 phage was dissolved in a wash buffer (tris-buffered saline supplemented with 0.5% Triton X-100), and applied to the well plate. The plate was incubated for 1 h at 25 °C with shaking by using a maximizer (MBR-022UP, Taitec, Saitama, Japan), and then washed three times with the same buffer. The bound phage was incubated with T7 tail fiber monoclonal antibody (1:5,000 dilution; Merck Millipore, cat No. 71530-3) and anti-mouse IgG HRP-linked antibody (1:5,000 dilution; Cell Signaling, cat No. 7076) for 1 h at 25 °C with shaking. After washing, *O*-phenylenediamine dihydrochloride substrate (SigmaFast OPD; Sigma Aldrich, cat No. P9187) was added, and the absorbance was quantified using a microplate reader equipped with a 450 nm band-pass filter (Bio-Rad).

### 3.8. Peptide Synthesis

An *unmodified* fluorescent peptide (H_2_N-K-5/6-FAM-A-M-W-C-Q-Y-H-P-Q-N-C-Y-K-M-NH_2_) was prepared by solid phase peptide synthesis using a semi-automated personal synthesizer (PetiSyzer®; HiPep Laboratories, Kyoto, Japan) as described previously [30]. Fmoc amino acids and Fmoc-Lys(5/6-FAM) were purchased from HiPep Laboratories (Kyoto, Japan) and AAT Bioquest (Sunnyvale, CA, USA), respectively. The solid phase synthesis was performed on Fmoc-NH-SAL-PEG resin (cat No. A00213, Watanabe Chemical Industries, Hiroshima, Japan). Chain elongation was carried out by using 1-Hydroxy-7-azabenzotriazole (HOAt) and *N,N,N',N'*-tetramethyl-O-(7-azabenzo-triazol-1-yl)uronium hexafluorophosphate (HATU) as the coupling reagent in the presence of diisopropylethylamine (DIPEA)/*N,N*-dimethylformamide (DMF)/N-methylpryrrolidone (NMP). The coupling was set to 50 °C for 40 min. The resin-bound peptide was cleaved by trifluoroacetic acid (TFA)/water/triisopropylsilane (TIS)/1,2-ethandithiol (EDT) (94/2.5/1/2.5 v/v/v/v) for 1 h at 50 °C. The crude peptide was dissolved in 0.1% formic acid (aq) and then purified with reverse-phase HPLC (Shimadzu, Kyoto, Japan) equipped with XTerra Prep MS C_18_ column (10 × 50 mm, Waters., Milford, MA, USA) and XBridge Prep C_18_ column (10 × 50 mm, Waters). The peptide was separated using a 0%–100% linear gradient of acetonitrile containing 0.1% formic acid during 12 min at a flow rate of 4 mL per minute. Characterization of the peptide was performed by LC-MS (Supplementary Figure S2). Purity was estimated to be above 95%. For the conjugation of Sal, the neutralized alkylating reagent (Sal-IA-NaOH) and neutralized TCEP (pH 7) were mixed with each peptide (100 μM) in a phosphate buffer (20 mM phosphate-KOH, pH 7.4) at final concentrations of 1 mM and 500 μM, respectively. The mixture was incubated overnight at 37 °C in the dark with shaking. After addition of formic acid at a final concentration of 2%, the peptide was purified with reverse-phase HPLC, and then the purified peptide was characterized by LC-MS (Supplementary Figure S2). Purity was estimated to be above 90%.

### 3.9. Fluorescence Polarization Assay

Fluorescence polarization was measured with a HYBRID-3000ES (Photoscience, Tokyo, Japan) equipped with appropriate filters (Ex. 480 nm and Em. 535/40 nm). The Sal-conjugated fluorescent peptide (4 pmol) was incubated with various concentrations of streptavidin, NeutrAvidin, or bovine serum albumin (BSA) in tris-buffered saline (50 mM Tris-HCl, pH 8.0, 150 mM NaCl) supplemented with 1 mM TCEP-NaOH at 30 °C. Concentration of the fluorescent peptide was determined by absorption coefficient at 495 nm. NeutrAvidin® and BSA were purchased from Thermo Scientific and Nacalai tesque, respectively. Concentrations of streptavidin and NeutrAvidin were determined by absorption coefficient at 280 nm. Concentration of BSA was determined by Bradford protein assay (Bio-Rad). Klotz plot was generated by GraphPad Prism software 6.0 (GraphPad Software, San Diego, CA, USA), and the sigmoid curve was fitted with non-linear least squares analysis to obtain the dissociation constant. For the negative control experiment, the Sal-*unmodified* fluorescent peptide (4 pmol) was used for the fluorescence polarization assay with the same procedure described above. For the competitive binding assay, various concentrations of biotin were added to the solution containing Sal-conjugated peptide-streptavidin complex (4 pmol of the peptide and 3 nmol of streptavidin; 20 nM and 15 μM, respectively). Biotin was purchased from New England Biolabs (Ipswich, MA, USA).

## 4. Conclusions

In conclusion, site-specific conjugation of haloacetamide derivatives to the designated Cys on bacteriophage T7-displayed peptides was achieved. This took place almost quantitatively without side reactions. The structure of the modified peptide on phage was identified by tandem mass spectrometry, and the conjugation yield was estimated by SDS-PAGE followed by fluorescence imaging in a rapid and universal manner. Generation of a novel pharmacophore by conjugation of a drug-like core molecule to fully randomized peptide on T7 phage was also demonstrated. Recently, optimization of a pharmacophore by conjugation of a core molecule with a library peptide via the mRNA-display and M13-phage display techniques were reported [9,31]. In both cases, the core molecule has already been known to bind to a target protein, and the binding ability was *improved* by *in vitro* selection. In contrast, we demonstrated that even optimization of a small drug-like core molecule, which is *never* known to bind to a target protein, could generate a novel pharmacophore. We envision that computer assisted *de novo* designing [32], data mining from broad public databases [33], and/or docking simulation of the small drug-like core molecule [33,34] followed by optimization of its surroundings by peptide via the 10BASE_d_-T will be a general technology for drug discovery. 

## Supplementary Materials

Supplementary materials can be accessed at: http://www.mdpi.com/1420-3049/19/2/2481/s1.
