# Supplementary material for: Pharmacophore Generation from a Drug-like Core Molecule Surrounded by a Library Peptide via the 10BASEd-T on Bacteriophage T7
Source: Molecules. 2014 Feb 21;19(2):2481–96. doi: 10.3390/molecules19022481 (PMC6271298; doi:10.3390/molecules19022481)

Supplementary Information

Figure S1. Identification of peptide fragments derived from fluorescein (Flu)-conjugated T7 phage-displayed peptide by LC-MS/MS. Upper panel: 450 nm (Flu absorption under low pH condition) chromatogram of the trypsinized peptide-fused gp10. Note that trypsin could not cleavage before proline. Middle and lower panels: MS and MS/MS spectra correspond to the trypsinized two peptide fragments possessing a Flu moiety.

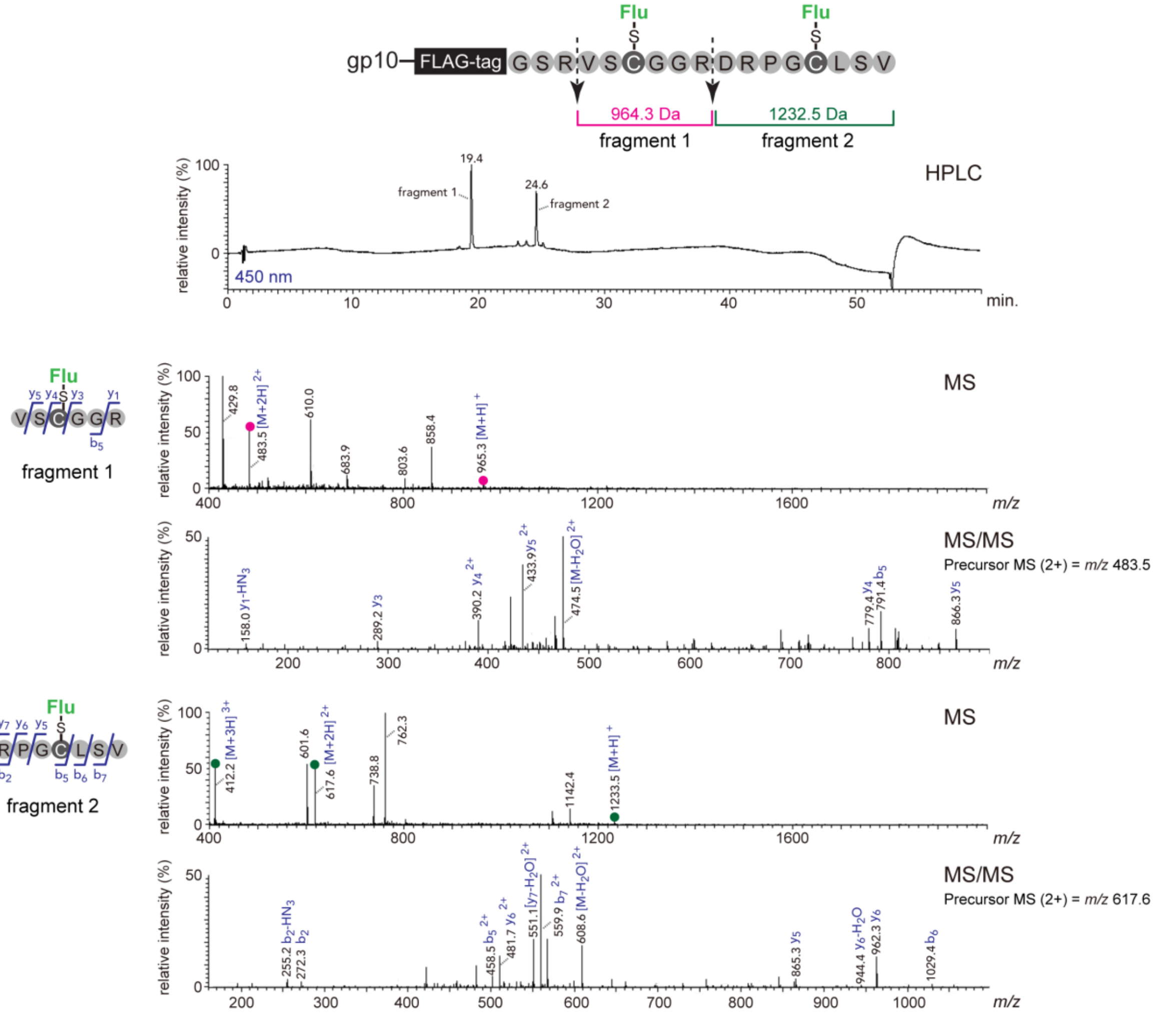

Figure S2. Mass spectrometric analysis of chemically synthesized peptides. MS spectra of the mock peptide (upper panel) and the Sal-conjugated peptide (lower panel).

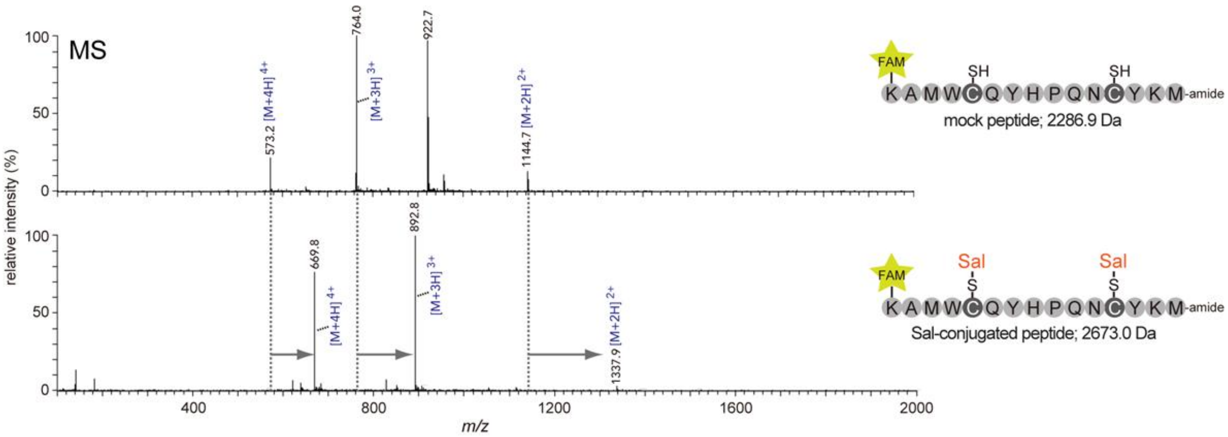

Supplement: Supplementary file 1 [file molecules-19-02481-s001.pdf]
